# Opinion Formation Models on a Gradient

**DOI:** 10.1371/journal.pone.0114088

**Published:** 2014-12-04

**Authors:** Michael T. Gastner, Nikolitsa Markou, Gunnar Pruessner, Moez Draief

**Affiliations:** 1 Institute of Technical Physics and Materials Science, Research Centre for Natural Sciences, Hungarian Academy of Sciences, Budapest, Hungary; 2 Department of Engineering Mathematics, University of Bristol, Bristol, United Kingdom; 3 Department of Mathematics, Imperial College London, London, United Kingdom; 4 Department of Electrical and Electronic Engineering, Imperial College London, London, United Kingdom; University of Namur, Belgium

## Abstract

Statistical physicists have become interested in models of collective social behavior such as opinion formation, where individuals change their inherently preferred opinion if their friends disagree. Real preferences often depend on regional cultural differences, which we model here as a spatial gradient *g* in the initial opinion. The gradient does not only add reality to the model. It can also reveal that opinion clusters in two dimensions are typically in the standard (i.e., independent) percolation universality class, thus settling a recent controversy about a non-consensus model. However, using analytical and numerical tools, we also present a model where the width of the transition between opinions scales 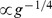, not 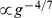 as in independent percolation, and the cluster size distribution is consistent with first-order percolation.

## Introduction

Disagreement between neighbors costs energy, in human societies as well as in ferromagnetic spin interactions. Because of this similarity, statistical physicists have recently shown great interest in models of opinion formation (e.g. [1]–[6], see [7], [8] for literature reviews). Individual actors in a population are regarded as nodes in a network and their opinions represent political affiliations, religions or consumer choices (Microsoft Windows vs. UN*X, Blu-ray vs. HD-DVD, etc.). The nodes influence each other's opinions along the edges in the network according to rules specific to the model in question. Rules that allow a critical mass of like-minded peers to persuade a disagreeing individual have recently found support in behavioral experiments [9]. The resulting opinion dynamics has been linked to election outcomes [10], [11] and innovation diffusion [12], [13], suggesting lessons for political campaigns [14] and advertisement [15].

Many opinion formation models embedded in two-dimensional space have only one stable solution, namely complete consensus [3], [5], [16], in particular when they implement deterministic rules. In reality, however, deterministic social behavior and perfect agreement are rare [17] – at least one small village of indomitable Gauls always holds out against the Romans. Some models thus allow clusters of a minority opinion to persist even if entirely surrounded by the opposite opinion [18], [19]. In this case, percolation theory provides the tools to analyze the geometry of the minority clusters [19], [20]. However, the results [19], [21] have been subject to some controversy because long-range correlations, thought to be responsible for deviations from independent percolation, are expected to require a long time to develop from an uncorrelated initial state [22]. Clearly, interactions generate complex correlations that can obscure the familiar scaling behavior of independent percolation. However, as illustrated in the present work, one must exercise great care before concluding that a given interaction spoils the (asymptotic) scaling of independent percolation.

In this article we tackle the open question: can opinion dynamics, with or without a stochastic element, fundamentally alter percolation properties such as the clusters' fractal dimensions or the cluster size distribution? We show that in many cases we retrieve the scaling laws of independent percolation. Moreover, we also give one example where a slight change of the dynamic rules leads to a radically different scaling behavior.

## Methods

We focus on models where the nodes are placed on a square lattice with edges linking them to their four nearest neighbors. Each node holds one of two possible opinions: “black” or “white”. Initially, the probability to be black is independent at all sites and given by
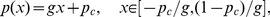
(1)where *x* is the node's horizontal position and 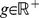 a constant gradient. (We set the intercept *p_c_* equal to the percolation threshold for later convenience.) We interpret *p*(*x*) as the innate propensity to hold the black opinion at the beginning as well as during the evolution of the opinions. Thus, nodes on the far left and far right of the lattice are likely to have opposite opinions. Some previous spatial models have included heterogeneous agents [23]–[25], but no gradient. In contrast, election results in various countries exhibit clear, smooth gradients, especially between progressive urban and conservative rural areas [26]–[28]. Our model resembles such a “culture war” fought on a gradient.

Including a non-zero gradient in the numerical simulations also has advantages for studying percolation properties [29]. As opposed to running many individual simulations for a range of different values of *p*, a gradient model allows us to analyze, in a single simulation, clusters for a whole interval of *p* rather than a single fixed value.

In the present work we consider opinion formation according to the following local rules.

- Majority vote (MV): the node follows the majority opinion of its four nearest neighbors. If both opinions are equally represented, no opinion change occurs.
- Unanimity rule (UR): the node changes its current opinion if and only if all of its nearest neighbors hold the opposite opinion [30].
- Independent percolation (IP): the node keeps its current opinion irrespective of the surrounding opinions.

When a node is updated, it follows the local rule with probability *q*. Otherwise it independently chooses a random opinion according to Eq. 1, so that 1−*q* is the level of noise entering the dynamics. Notably, Eq. 1 is the only way for the local prevalence of a certain opinion and thus the gradient to enter into the dynamics of the system. At *q* = 1 the evolution is affected by the presence of the gradient only through the initial condition. At *q*<1 the random updates during the evolution exhibit the innate propensity gradient towards one or the other opinion by allowing agents to revert to their original opinion even if it contradicts the local majority.

All nodes simultaneously update their opinion at each time step, but other choices such as random sequential updates do not change our findings noticeably. The latter may have the more immediate social interpretation as an ongoing opinion formation with agents re-considering choices with a fixed rate, but simultaneous updates are, surprisingly, slightly more accessible analytically. For a fixed value of *q*, we abbreviate the models by MV*_q_* or UR*_q_*, respectively. We do not need a subscript *q* for IP because, regardless of the value of *q*, any snapshot of the lattice looks statistically alike, depending only on the parameters *p_c_* and *g* in Eq. 1.

**Figure 1:**
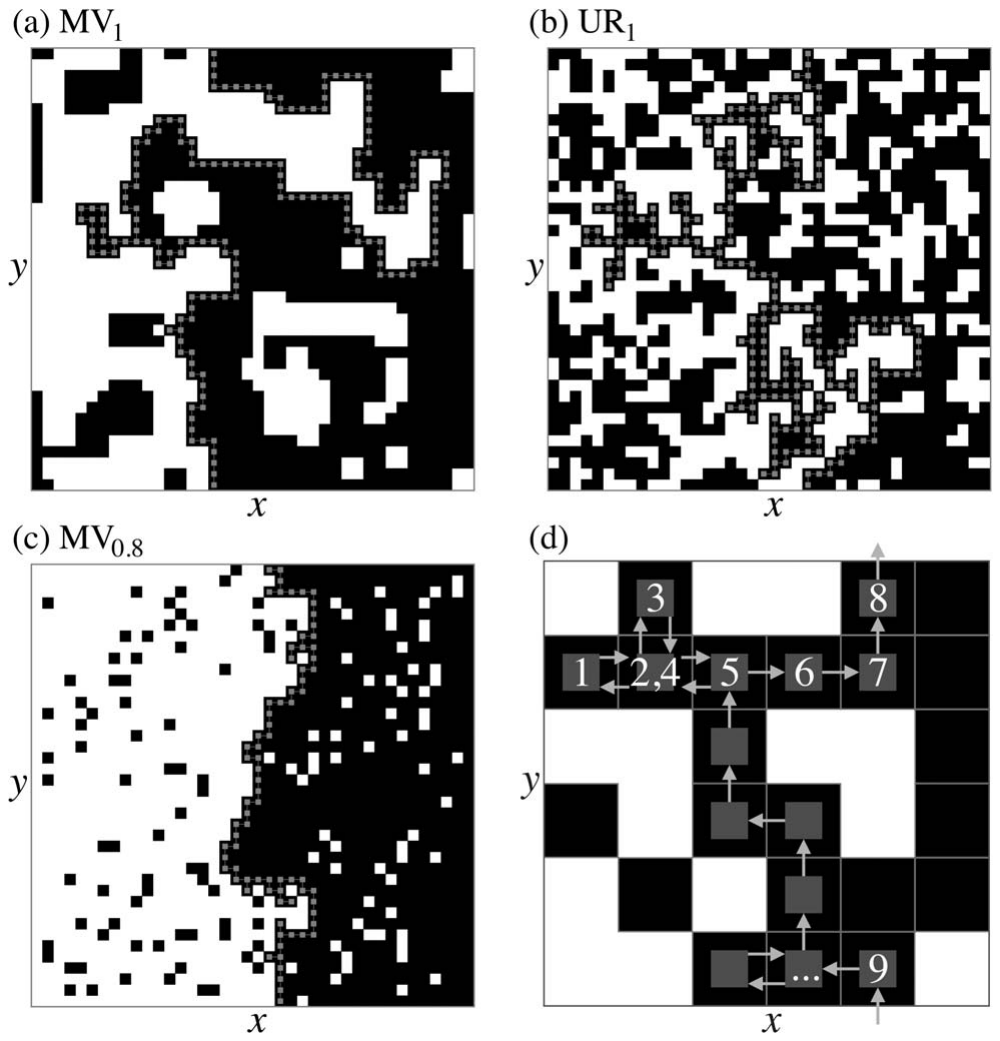
Opinion distributions and percolation hull. We show typical steady-state opinion distributions for *g* = 5×10^−3^ and (a) MV_1_, (b) UR_1_, (c) MV_0.8_. The two opposing opinions are shown as black and white squares. The sites marked by gray squares form the spanning cluster's hull. (d) Illustration how the hull can be parameterized by a left-turning walk [33].

Once the model reaches the steady state, we study the geometric properties of the clusters formed. On the left of Fig. 1(a)–(c), the black clusters form small isolated islands, whereas on the right a single large black cluster spans from top to bottom [31]. This percolation transition can be characterized by the hull of the spanning cluster [32], defined as the following left-turning walk [33], [34]. We start the walk at a site with minimal *x*-coordinate in the black spanning cluster and face towards the right (Fig. 1d). First we attempt to turn to the neighbor on our left, but step in this direction only if we reach a black site. Otherwise, we try to move forward, then to the right, and finally backward until we have discovered the first black neighbor. If we iterate this procedure and apply periodic boundary conditions in the *y*-direction, the hull has visited the entire front of the spanning cluster when it returns to the starting position.

## Results and Discussion

Our numerical and analytical findings are summarized in Table 1. In the following we discuss them in detail.

**Table 1:** Summary.

Model	*q*	Exponents	Universality Class
Independent Percolation (IP)		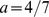 , 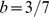 , 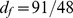 , 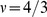	IP (by definition)
Deterministic Majority Vote Model (MV_1_)	1	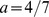 , 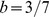 , 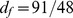 , 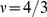	IP
Deterministic Unanimity Rule (UR_1_)	1	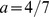 , 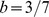 , 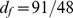 , 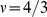	IP
Stochastic Majority Vote Model (MV_0.8_)	0.8	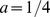 , 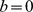 , 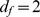	Edwards-Wilkinson
Stochastic Unanimity Rule (UR_0.8_)	0.8	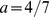 , 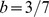 , 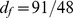 , 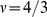	IP

Summary of the results. For definitions of models and exponents see text.

### Steady-state hull width and length

If *q* = 1, the dynamics is deterministic and the only source of randomness lies in the initial assignment of opinions. In this special case, MV_1_ is identical to the non-consensus opinion model of Ref. [19], where it was already noted that a small fraction of the nodes – in our simulations 1.2% on average at *p_c_* = 0.50643(1) – keeps switching opinions with period 2. When all other nodes have stopped changing opinions, we will consider MV_1_ to have reached its steady state. The convergence is quick: a non-periodic node freezes after a mean of only 0.8 time steps. In UR_1_, oscillatory opinions can occur only if the initial opinions form a perfect checkerboard pattern. Because the gradient pins the left (right) edge to be entirely white (black), a checkerboard pattern is impossible. Hence, every node reaches a stationary opinion, on average after just 0.06 updates at *p_c_* = 0.549199(5). For IP, percolation occurs, as in zero-gradient percolation, at *p_c_* = 0.59274(1) [35].

If *q*<1, the opinions in MV*_q_* and UR*_q_* never freeze, but, after a transient, the stochastic time series of black occupancy in any column *x* becomes stationary. All measurements for *q*<1 presented here were made at *q* = 0.8 in this steady state. A visual comparison between Fig. 1(a)–(c) suggests a qualitative difference between MV_1_ and UR_1_ on the one hand and MV_0.8_ on the other hand. In the latter case, the spanning cluster appears significantly more compact and the hull, which is centered at *p_c_* = 0.5000(4), much straighter. So, counterintuitively, the stochastic dynamics of MV_0.8_ anneals rather than roughens the surface compared to MV_1_ and UR_1_.

We can quantify this observation by computing the hull's width *w* and length *l*. If the hull consists of the walk 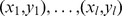, we define
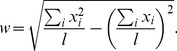
(2)


**Figure 2:**
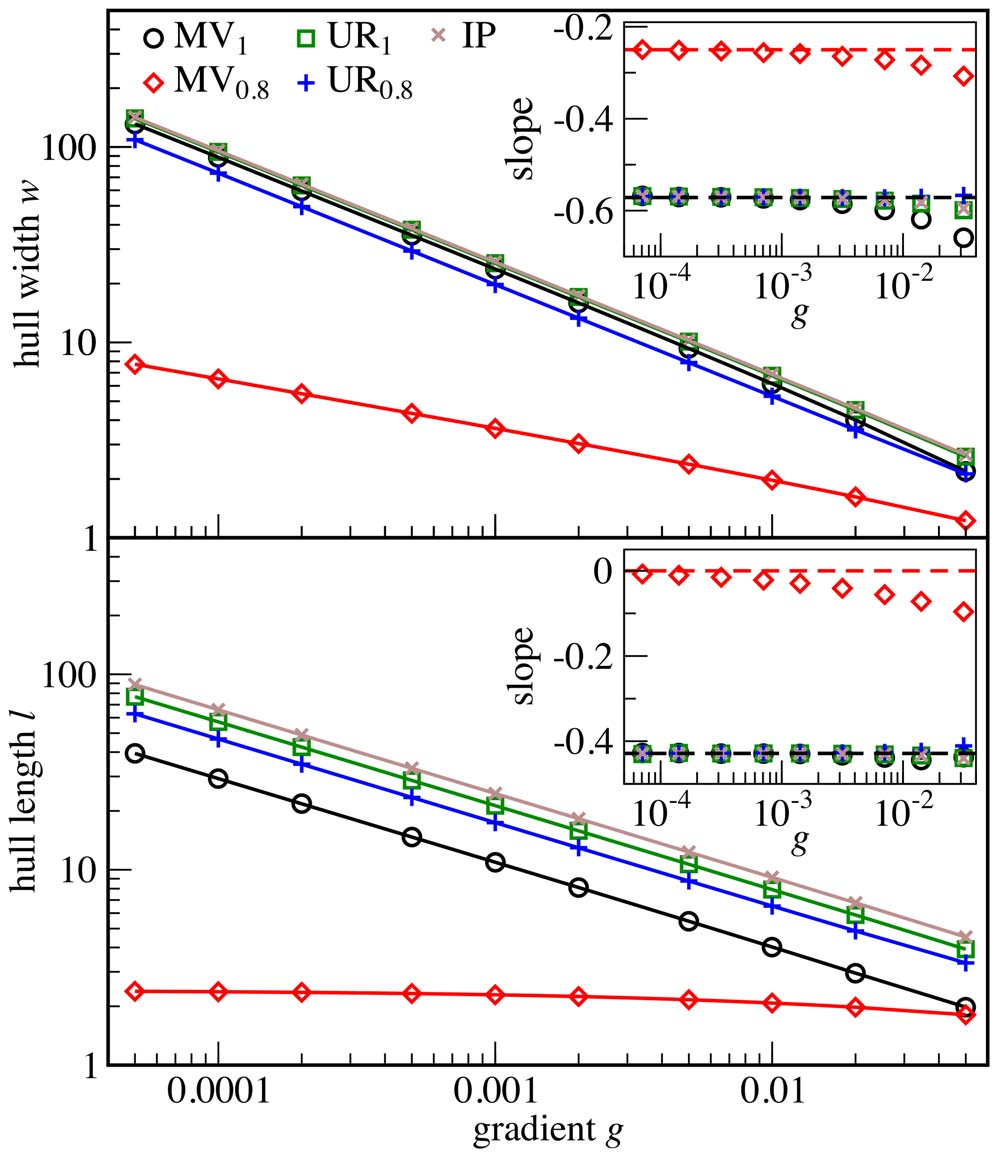
Mean hull width and length determined numerically as a function of the gradient. Insets: slope in doubly-logarithmic scales (i.e. 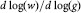 in upper, 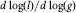 in lower panel). Dashed lines indicate the limiting slopes for 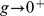 which follow from scaling analysis (see text): −4/7 and −1/4 in the upper, −3/7 and 0 in the lower panel. Error bars are smaller than the symbol sizes.

As the numerical results in Fig. 2 show, the width and length for all models scale as power laws 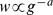 and 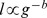 in the limit 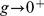. With only one exception among all investigated cases, the results are consistent with 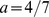 and 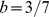, the exact exponents of independent gradient percolation [36]. We also retrieve the correlation length critical exponent 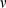 of standard percolation via the formula 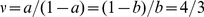 [31]. The notable exception is MV_0.8_ with 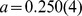 and 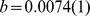, based on numerics for 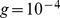 and 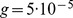. Studying the dependence of 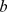 on *g* systematically suggests 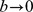 for 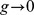, while 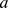 stays close to 1/4. In fact, the analytical results presented below indicate that 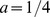 and 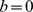. In independent percolation, 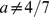 can arise only if the probability to be black increases nonlinearly at the percolation threshold [37]. However, in that case the ratio 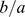 must still equal 3/4 which is not true for MV_0.8_ so that we must look elsewhere for an explanation.

We will briefly summarize why 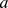 equals 1/4 for MV*_q_* if 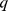 is close to, but not equal to 1. For details we refer to the online Information S1. We make two approximations. (1) The hull can be treated as a single-valued function of 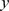 so that we can parameterize the hull at time 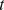 as a function 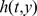. (2) In MV_0.8_, as opposed to UR*_q_* and IP, we observe only few isolated minority nodes, which motivates a “solid-on-solid” approximation: we neglect that there is a small number of black (white) sites to the left (right) of 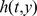. With the notation 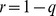, the only transition probabilities for 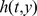 up to terms of order 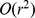 are (see Information S1)
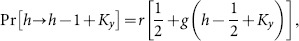
(3)

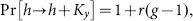
(4)

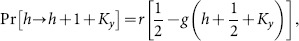
(5)where 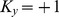 if 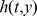 is a strict local minimum in *y*, 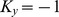 for a maximum, and 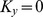 otherwise. In the continuum limit [38], the leading terms in the evolution of the hull are (see Information S1)
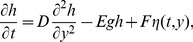
(6)where 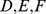 are independent of *g* and 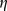 is white noise with mean zero and covariance 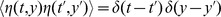. Equation 6 is the Edwards-Wilkinson equation [39] with an Ornstein-Uhlenbeck restoring force [40], [41] and can be integrated (see Information S1) to obtain the continuum limit of Eq. 2,
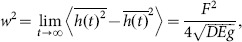
(7)where the angle brackets denote the ensemble average and the overlines symbolize spatial averages. Thus, we obtain 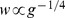 consistent with the numerical results for MV_0.8_. Although we have here derived the scaling law only for the MV model, numerical evidence suggests that 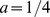 is valid for a broader class of gradient models. In Ref. [42], a numerical fit for a spatial birth-death process on a gradient also yields 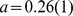.

### Cluster sizes

The scaling laws for *w* and 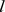 signal that MV_0.8_ is not in the same universality class as IP. In Ref. [19] it is claimed that MV_1_ is in yet another class, namely invasion percolation with trapping (IPT). Although *w* scales identically in IP and IPT [43], we now demonstrate how the gradient method can still show unequivocally that MV_1_ belongs to the IP class after all, thus supporting the arguments of Ref. [22]. We calculate the size *s*_max_ of the largest cluster in a lattice whose linear size is *L* in both *x*- and *y*-direction. We center the *x*-axis at *p_c_* so that the initial probability to be black in Eq. 1 is limited by 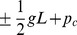 on the right (left) edge. As a function of *L* and *g*, *s*_max_ is expected to satisfy the ansatz
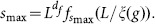
(8)


**Figure 3:**
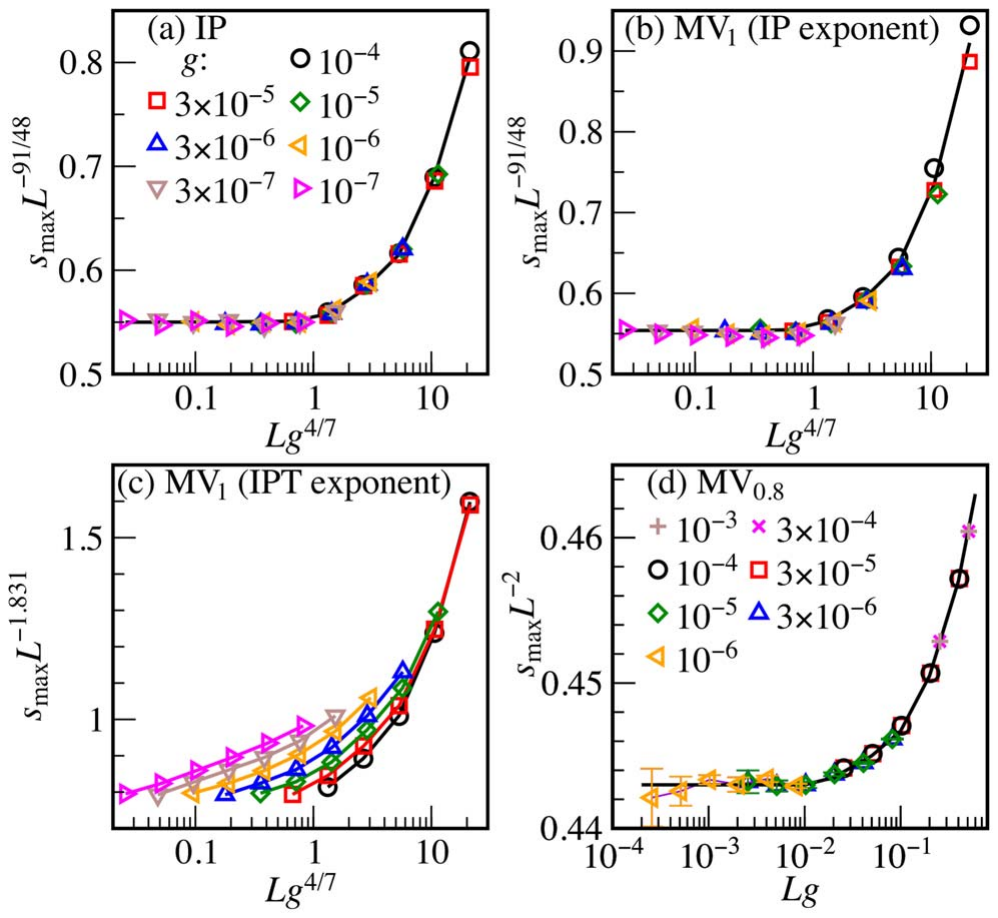
Fractal dimensions. For the correct exponents 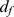 and *c*, 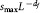 as a function of 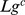 should collapse on a single curve with slope zero for 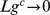. For (a) IP and (b) MV_1_, 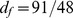 is the same as the fractal dimension of standard percolation. (c) Replacing 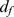 with the value 1.831 of invasion percolation with trapping (IPT) does not produce a data collapse. (d) For the largest MV_0.8_ cluster, we obtain a data collapse if 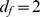.

Here 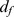 is the fractal dimension of the cluster at 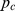, 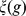 is the characteristic length scale for changes in the cluster density, and the scaling function 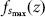 approaches a constant for 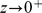. The fractal dimensions differ between the two universality classes in question: 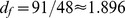 for IP and 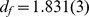 for IPT [44]. Furthermore, 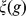 in IP scales linearly with 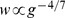 [31]. Thus, according to Eq. 8, a plot of 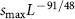 versus 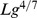 collapses the IP data for different *L* and *g* on a single curve that asymptotically approaches a constant for small 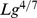 (Fig. 3a). For MV_1_, we obtain a data collapse with the same IP exponents (Fig. 3b). By contrast, if we assume 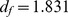, there is neither a collapse nor do the individual curves approach a constant for 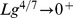 (Fig. 3c), hence ruling out that MV_1_ is in the same universality class as IPT. Changing the exponent 4/7 on *g* leads to a lateral shift of the data in Fig. 3(c), but we found no value yielding a convincing data collapse. Moreover, it cannot overcome the problem that the hypothetical scaling function 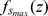 would not become constant for 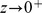. However, the collapse of MV_0.8_ with 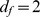 (which lends further support to the solid-on-solid approximation) and 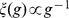 in Fig. 3(d) corroborates that opinion dynamics can lead to percolation outside the IP universality class.

**Figure 4:**
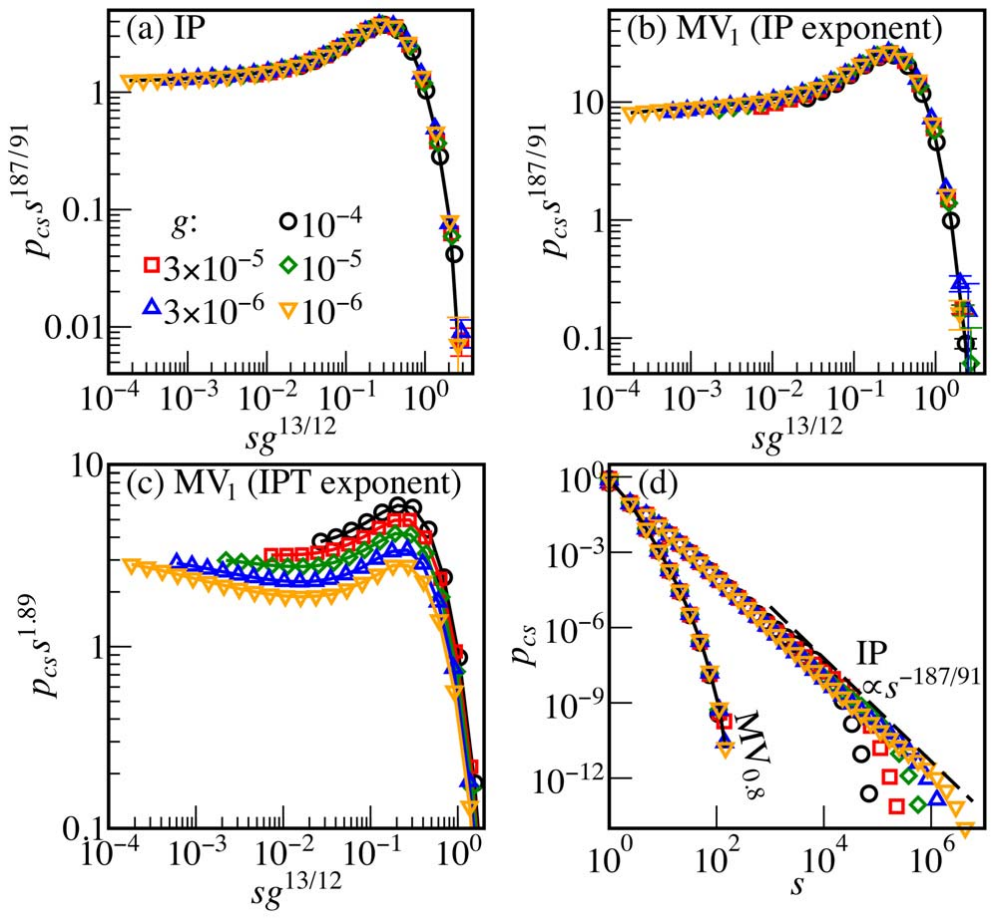
Cluster size distributions. (a) The rescaled distribution 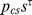 for IP collapses if plotted versus 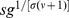, where the critical exponents 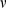, 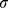, 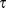 are those of standard percolation. For MV_1_ the data collapse is much better (b) for the IP exponent 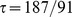 than (c) for the IPT exponent 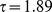. (d) The MV_0.8_ distribution does not follow the same asymptotic power law as IP.

The cluster size distribution provides further support for this classification. We count all non-spanning clusters with at least one site in the stripe 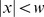 and compute the fraction 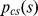 of clusters of size *s*. In IP [42]

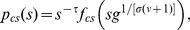
(9)where the critical exponents are 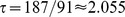, 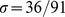, 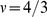 [45], and 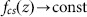 for 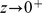 (Fig. 4a). Reference [19] hypothesizes that in MV_1_ the exponent 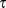 is replaced by 1.89(1), the corresponding value for the pore size distribution in IPT. However, Fig. 4(b) and (c) show that, while the data collapse is excellent for 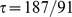, it is poor for the alternative value 1.89. In summary, MV_1_ and IP share the following critical exponents: the hull width and length exponents *a*, *b* and consequently 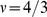; the fractal dimension 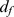 and thus 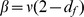; furthermore 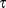 and 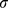. This list is clear evidence that MV_1_ is in the IP universality class. As shown in the Information S1, we reach the same conclusion for UR_1_ and UR_0.8_.

The situation is different in MV_0.8_ where the cluster size distribution appears to drop more sharply with a cutoff that varies much less with the gradient. We want to assess the lack of scaling quantitatively and distinguish it from a power law with large exponent 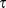 and little dependence of the upper cutoff on *g*. Moment ratios 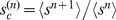 are asymptotically proportional to the upper cutoff, provided 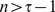. If the transition is continuous, then 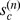 scales asymptotically as a power of *g*. This power law can be detected more easily than the asymptotic scaling regime 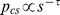 [46].

**Figure 5:**
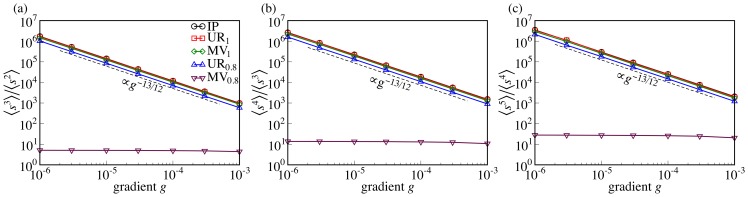
Cluster size moment ratios. The moment ratios 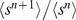 of the cluster size distributions for (a) 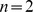, (b) 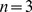, (c) 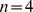. The ratios for UR_1_, MV_1_, and UR_0.8_ scale in the same manner as in IP, namely 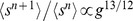. By contrast, the moment ratios for MV_0.8_ appear to reach an asymptotic limit for 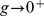.

We plot the moment ratios of IP, UR_1_, MV_1_, UR_0.8_ and MV_0.8_ for 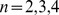 in Fig. 5. Except MV_0.8_, all of these cases are in excellent agreement with the prediction of Eq. 9, 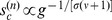, where 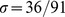 and 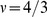 are the critical exponents of IP [45]. The cutoff 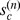 in MV_0.8_, by contrast, does not diverge as a power law for 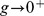. Instead 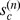 appears to reach an asymptotic value for all *n*. Such a behavior is typical of a first-order transition. Based on these data, we can firmly rule out that 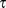 in MV_0.8_ has the IP value 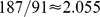. We add the caveat that, for sufficiently large *n*, 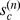 may scale as a power of *g* after all. However, the data imply 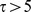, an unusually large value compared to IP, directed percolation (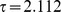) [47] and Achlioptas percolation (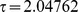) [48].

## Conclusions

We have studied in total five opinion dynamics models on a gradient, as summarized in Table 1. One of the models we studied, independent percolation, provides the very definition of the corresponding universality class, IP. We find that of the four other models studied, three display features that are fully compatible with IP, which is commonly observed in gradient models with and without interaction [29], [49], [50].

One model, MV_0.8_, differs from all of the above. At 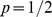 it has states with either a black or white majority. Without a gradient, (i.e. 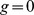 in Eq. 1, so that 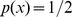 is constant in *x*), there are two stable stationary solutions, where one state is above and the other below the threshold of percolation of, say, black sites. There is hysteresis if one tries to move from one majority to the other by tuning *p*, as expected for first order transitions. By introducing a gradient, the two phases are forced to collide because the left boundary must be completely white and the right boundary black. We observe that the gradient stabilizes and sharpens the front compared to independent percolation.

MV_0.8_ differs from the other models in two important points. First, its stochastic nature helps anneal boundaries between opposite opinions. The second difference is that the majority rule makes small clusters more prone to invasion by the opposing opinion. The combination of these two features results in what appears to be a first order transition. Nevertheless, the opinion interface displays scaling, found to be in the Edwards-Wilkinson universality class, which differs significantly from independent percolation.

The birth-death model of Ref. [42] suggested already the possibility of first-order transitions in gradient models. We leave it to future research to analytically confirm the first-order nature of the MV_0.8_ transition. It would also be insightful to investigate more complex network topologies that are based on real social interactions rather than a regular square lattice. We emphasize that, in the light of previous work on explosive percolation [48], [51]–[53], only analytic results can fully clarify the order of any percolation transition. However, we can conclude with certainty that, although none of the opinion models we have investigated is consistent with IPT, MV_0.8_ is an example of a dynamic rule that leads to percolation outside the IP universality class.

From a sociological perspective, our study shows that small variations in the innate propensity towards one or another opinion may turn into a spatial discontinuity in the opinions. Interestingly, the sharpest division occurs when agents do not follow the local majority all the time. Hence, processes that may be perceived as having the effect of making the interface between different opinions more blurred, such as the majority rule with stochasticity involved, have the opposite effect. They anneal that interface and contribute to the collapse of minority clusters, which are sustained in the presence of stricter rules, such as the deterministic unanimity rule.

## Supporting Information

Information S1 **Derivation of Eq. 3–7 and data showing that UR_1_ and UR_0.8_ are in the IP universality class.**
(PDF)
