# Supplementary material for: Opinion Formation Models on a Gradient
Source: PLoS One. 2014 Dec 4;9(12):e114088. doi: 10.1371/journal.pone.0114088 (PMC4256392; doi:10.1371/journal.pone.0114088)
Supplement: Information S1 — Derivation of Eq. 3–7 and data showing that UR1 and UR0.8 are in the IP universality class. (PDF) [file pone.0114088.s001.pdf]

# Supplementary information for the article: "Opinion formation models on a gradient"

Michael T. Gastner, Nikolitsa Markou, Gunnar Pruessner, and Moez Draief

## 1 Transition probabilities in the hull dynamics of the majority vote model (Eq. 3–5 in main text)

Let the hull be parameterized as $(x_1, y_1), \dots, (x_l, y_l)$ by the left-turning walk described in the main text. Figure 1c of the main text shows that the hull in $\mathrm{MV}_{0.8}$ separates a predominantly white region on the left from a similarly dense black region on the right. This observation justifies a "solid-on-solid" approximation [1] where we ignore

- any overhangs in the interface (i.e., parts of the left-turning walk that move towards smaller $y$-coordinates),
- any isolated islands of the minority color to the left and right of the hull.

In this approximation, the hull at time step $t$ is completely characterized by

$$h(t, y) = \min\{x_k | y_k = y, k = 1, \dots, l\} - \frac{1}{2} \tag{1}$$

because every site $(x, y)$ with $x < h(t, y)$ will be white and every site with $x > h(t, y)$ black. We now have to distinguish three cases.

### 1.1 Case 1: $h(t, y)$ is neither a strict local minimum nor maximum

In this case, one of the following four conditions must be met

- $h(t, y - 1) = h(t, y)$,
- $h(t, y) = h(t, y + 1)$,
- $h(t, y - 1) < h(t, y) < h(t, y + 1)$, or
- $h(t, y - 1) > h(t, y) > h(t, y + 1)$.

Let $A$ be the white site in the $y$-th row with $x$-coordinate $h(t, y) - \frac{1}{2}$ and $B$ the black site at $h(t, y) + \frac{1}{2}$ (see Fig. 1). In all cases listed above, both $A$ and $B$ have at least two neighbors of their own color, namely one in the $y$-th row and one in a neighboring row. Including their own vote, the local majority supports their current opinion. As a consequence, in the deterministic majority vote model $\mathrm{MV}_1$ neither $A$ nor $B$ will change color and thus $h(t + 1, y) = h(t, y)$. In the stochastic model $\mathrm{MV}_{1-r}$ with $r > 0$, the probability that $A$ becomes black is

$$\Pr[A \text{ black at } t + 1 \,|\, h(t, y) = x] = r \left[ g\left(x - \frac{1}{2}\right) + p_c \right], \tag{2}$$

and the probability that $B$ becomes white

$$\Pr[B \text{ white at } t + 1 \,|\, h(t, y) = x] = r \left[ 1 - g\left(x + \frac{1}{2}\right) - p_c \right]. \tag{3}$$

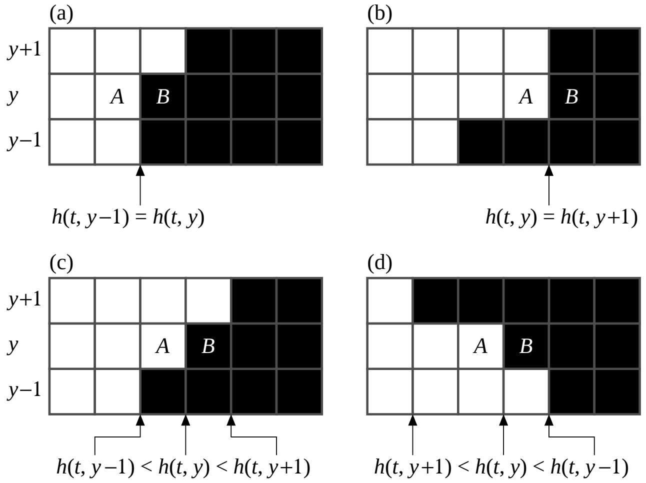


Figure 1: Examples where $h(t, y)$ is neither a strict local minimum nor maximum. In each case, the white and black sites at the interface, $A$ and $B$, have at least two neighbors of their own color so that in $MV_1$ there is no change of the hull position (i.e., $h(t, y) = h(t+1, y)$). In $MV_{1-r}$, there is a $O(r)$ probability that the hull moves one site to the left or right. All other probabilities are $O(r^2)$.

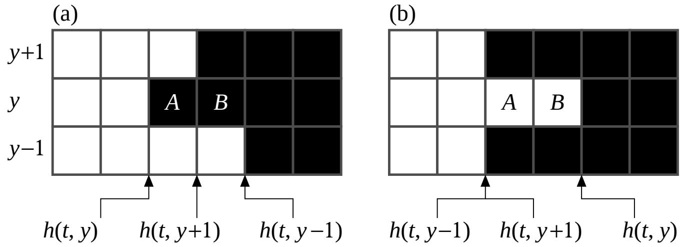


Figure 2: Examples where $h(t, y)$ is a strict local (a) minimum or (b) maximum. The minimal or maximal site of the protruding opinion is in a local minority, but all other sites in row $y$ will have at least two neighbors of the same opinion. Thus, in $MV_1$ only the front site will change opinion between time steps $t$ and $t + 1$. In $MV_{1-r}$ the probability of the hull shifting one step towards the (a) right, (b) left is $1 - O(r)$. The probability of no change or two steps to the (a) right, (b) left is $O(r)$. All other transitions have probabilities $O(r^2)$.

In the solid-on-solid approximation, the hull can shift exactly one step to the left only if $A$ turns black, while all other sites keep their colors with a probability $1 - O(r)$. Because the probabilities are independent, we can multiply them and obtain

$$\Pr\left[h(t+1, y) = x - 1 \,|\, h(t, y) = x\right] = r\left[g\left(x - \frac{1}{2}\right) + p_c\right] + O(r^2). \tag{4}$$

Similarly,

$$\Pr[h(t+1, y) = x + 1 \,|\, h(t, y) = x] = r\left[1 - g\left(x + \frac{1}{2}\right) - p_c\right] + O(r^2). \tag{5}$$

Because shifts of more than one step to either side have probabilities $O(r^2)$ and the sum of the probabilities must equal one,

$$\Pr[h(t+1, y) = x \,|\, h(t, y) = x] = 1 + r(g - 1) + O(r^2). \tag{6}$$

## 1.2 Case 2: $h(t, y)$ is a strict local minimum

Here the leftmost black site $A$ in row $y$ is in a local minority (see Fig. 2a). It stays black with probability

$$\Pr[A \text{ black at } t+1 \,|\, h(t, y) = x] = r\left[g\left(x + \frac{1}{2}\right) + p_c\right]. \tag{7}$$

Its right neighbor $B$ is black, which is the local majority because at least its two neighbors in row $i$ are black. Hence, it becomes white with probability

$$\Pr[B \text{ white at } t+1 \,|\, h(t,y)=x] = r\left[1 - g\left(x+\frac{3}{2}\right) - p_c\right] \tag{8}$$

In the solid-on-solid approximation, the hull can only stay at the same position if none of the sites in row $i$ changes its color. With the only exception of $A$, an individual opinion change has probability $1 - O(r)$ for all sites so that

$$\Pr[h(t+1,y)=x \,|\, h(t,y)=x] = r\left[g\left(x+\frac{1}{2}\right) + p_c\right] + O(r^2). \tag{9}$$

The hull can only shift two sites to the right if $A$ and $B$ become white. The former has probability $1-O(r)$, the latter is given by Eq. 8, and all other probabilities are $1 - O(r)$. Therefore,

$$\Pr[h(t+1,y)=x+2 \,|\, h(t,y)=x] = r\left[1 - g\left(x+\frac{3}{2}\right) - p_c\right] + O(r^2). \tag{10}$$

All other shifts further to the left and right are $O(r^2)$, so that the only remaining transition of one step to the right has probability

$$\Pr[h(t+1,y)=x+1 \,|\, h(t,y)=x] = 1 + r(g-1) + O(r^2). \tag{11}$$

### 1.3 Case 3: $h(t,y)$ is a strict local maximum

In analogy to case 2, we find

$$\Pr[h(t+1,y)=x \,|\, h(t,y)=x] = r\left[1 - g\left(x-\frac{1}{2}\right) - p_c\right] + O(r^2), \tag{12}$$

$$\Pr[h(t+1,y)=x-2 \,|\, h(t,y)=x] = r\left[g\left(x-\frac{3}{2}\right) + p_c\right] + O(r^2), \tag{13}$$

$$\Pr[h(t+1,y)=x-1 \,|\, h(t,y)=x] = 1 + r(g-1) + O(r^2). \tag{14}$$

### 1.4 Summary

We can summarize the results so far with the notation

$$K_y = \begin{cases} +1 & \text{if } h(t,y) \text{ is a strict minimum,} \\ -1 & \text{if } h(t,y) \text{ is a strict maximum,} \\ 0 & \text{otherwise.} \end{cases} \tag{15}$$

Neglecting terms $O(r^2)$,

$$\Pr\left[h(t+1,y)=x-1+K_y \,|\, h(t,y)=x\right] = r\left[g\left(x-\frac{1}{2}\right) + p_c\right] + rgK_y, \tag{16}$$

$$\Pr\left[h(t+1,y)=x+K_y \,|\, h(t,y)=x\right] = 1 + r(g-1), \tag{17}$$

$$\Pr\left[h(t+1,y)=x+1+K_y \,|\, h(t,y)=x\right] = r\left[1 - g\left(x+\frac{1}{2}\right) - p_c\right] - rgK_y. \tag{18}$$

Because there are no isolated clusters in the solid-on-solid approximation and the dynamics is symmetric under interchange of black and white, $p_c$ equals $\frac{1}{2}$. This assumption is consistent with our numerical results for the full model $p_c = 0.5000(4)$. Equations 16–18 with $p_c = \frac{1}{2}$ yield Eq. 3–5 in the main text.

## 2 The stochastic differential equation for the hull evolution (Eq. 6 in main text)

An alternative formulation of Eq. 16–18 is

$$h(t+1,y) = h(t,y) + K_y + \zeta_y, \tag{19}$$

where

$$\Pr(\zeta_y = -1) = r\left[\frac{1}{2} + g\left(h(t,y) - \frac{1}{2} + K_y\right)\right], \tag{20}$$

$$\Pr(\zeta_y = 0) = 1 + r(g-1), \tag{21}$$

$$\Pr(\zeta_y = 1) = r\left[\frac{1}{2} - g\left(h(t,y) + \frac{1}{2} + K_y\right)\right]. \tag{22}$$

The expectation value of $\zeta_y$ is

$$\langle \zeta_y \rangle = -2gr(h + K_y), \tag{23}$$

so that we can rephrase Eq. 19 as

$$\underbrace{h(t+1,y) - h(t,y)}_{A} = K_y - \underbrace{2gr(h+K_y)}_{B} + \underbrace{\zeta_y - \langle \zeta_y \rangle}_{C}. \tag{24}$$

Our objective is to take the continuum limit of Eq. 24 in the following manner. With the notation

$$\Delta_+ = h(t, y+1) - h(t,y), \tag{25}$$

$$\Delta_- = h(t,y) - h(t, y-1), \tag{26}$$

we can express $K_y$ of Eq. 15 using the Heaviside step function

$$\theta(x) = \begin{cases} 1 & \text{if } x \geq 0, \\ 0 & \text{otherwise} \end{cases} \tag{27}$$

as

$$K_y = [1 - \theta(-\Delta_+)]\,[1 - \theta(\Delta_-)] - [1 - \theta(\Delta_+)]\,[1 - \theta(-\Delta_-)]. \tag{28}$$

The discontinuous Heaviside function can be written as the limit $\epsilon \to 0$ of the differentiable function [2]

$$\theta_\epsilon(x) = \epsilon \ln\left(\frac{\exp\left(\frac{x+1}{\epsilon}\right) + 1}{\exp\left(\frac{x}{\epsilon}\right) + 1}\right). \tag{29}$$

Simultaneously with the limit of the Heaviside function, we take the continuum limit of the space and time variables,

$$\tilde{t} = \epsilon^k t, \tag{30}$$

$$\tilde{y} = \epsilon^l y, \tag{31}$$

$$\tilde{h}(\tilde{t}, \tilde{y}) = \epsilon^m h(t,y), \tag{32}$$

with $k, l, m > 0$ and let $g$ approach zero as

$$g = \epsilon^n \tilde{g} \tag{33}$$

with $n > 0$. We will now determine the leading terms in the individual parts of Eq. 24, which will give us conditions for these exponents.

## 2.1 The discrete time derivative $A$ in Eq. 24

Assuming that $h$ is a smooth function, we can expand $A$ as

$$A = \epsilon^{-m}\left(\tilde{h}(\tilde{t} + \epsilon^k) + \tilde{h}(\tilde{t})\right) = \epsilon^{k-m}\frac{\partial \tilde{h}}{\partial \tilde{t}} + O\left(\epsilon^{2k-m}\right). \tag{34}$$

## 2.2 The variable $K_y$ encoding a strict minimum or maximum

For the derivatives of $\theta_\epsilon$ of Eq. 29, we find

$$\theta_\epsilon(0) = 1 - \epsilon\,\ln(2) + O\left(\epsilon\, e^{-1/\epsilon}\right), \tag{35}$$

$$\frac{d\theta_\epsilon(0)}{dx} = \frac{1}{2} + O\left(e^{-1/\epsilon}\right), \tag{36}$$

$$\frac{d^2\theta_\epsilon(0)}{dx^2} = -\frac{1}{4\epsilon} + O\left(\epsilon^{-1}e^{-1/\epsilon}\right), \tag{37}$$

$$\frac{d^3\theta_\epsilon(0)}{dx^3} = O\left(\epsilon^{-2}e^{-1/\epsilon}\right). \tag{38}$$

Inserting these derivatives into Eq. 28 we obtain

$$\begin{aligned} K_{y,\epsilon} = & \left[\epsilon\,\ln(2) + \frac{1}{2}\Delta_+ - \frac{1}{8\epsilon}\Delta_+^2 + O\left(\epsilon^{-3}\Delta_+^4\right)\right]\left[\epsilon\,\ln(2) - \frac{1}{2}\Delta_- - \frac{1}{8\epsilon}\Delta_-^2 + O\left(\epsilon^{-3}\Delta_-^4\right)\right] \\ & - \left[\epsilon\,\ln(2) + \frac{1}{2}\Delta_- - \frac{1}{8\epsilon}\Delta_-^2 + O\left(\epsilon^{-3}\Delta_-^4\right)\right]\left[\epsilon\,\ln(2) - \frac{1}{2}\Delta_+ - \frac{1}{8\epsilon}\Delta_+^2 + O\left(\epsilon^{-3}\Delta_+^4\right)\right] \\ & = \epsilon\,\ln(2)\left(\Delta_+ - \Delta_-\right) + \frac{1}{8\epsilon}\Delta_+\Delta_-\left(\Delta_+ - \Delta_-\right) + O\left(\epsilon^{-3}\Delta^5\right). \end{aligned} \tag{39}$$

From the Taylor expansions of $\tilde{h}$ we obtain

$$\Delta_+ - \Delta_- = \epsilon^{2l-m}\frac{\partial^2\tilde{h}}{\partial\tilde{y}^2} + O\left(\epsilon^{4l-m}\right), \tag{40}$$

$$\Delta_+\Delta_-\left(\Delta_+ - \Delta_-\right) = \epsilon^{4l-3m}\left(\frac{\partial\tilde{h}}{\partial\tilde{y}}\right)^2\frac{\partial^2\tilde{h}}{\partial\tilde{y}^2} + O\left(\epsilon^{6l-3m}\right), \tag{41}$$

so that

$$K_{y,\epsilon} = \epsilon^{2l-m+1}\ln(2)\frac{\partial^2\tilde{h}}{\partial\tilde{y}^2} + O\left(\epsilon^{4l-3m-1}\right), \tag{42}$$

where the expansion converges only if

$$l - m > 1. \tag{43}$$

## 2.3 The gradient term $B$ in Eq. 24

From Eq. 32, 33 and 42, we obtain

$$B = 2\epsilon^n\tilde{g}r\left[\epsilon^{-m}\tilde{h}(\tilde{t},\tilde{y}) + O\left(\epsilon^{2l-m+1}\right)\right] = 2\epsilon^{n-m}\tilde{g}r\tilde{h}(\tilde{t},\tilde{y}) + O\left(\epsilon^{2l-m+n+1}\right). \tag{44}$$

## 2.4 The noise term $C$ in Eq. 24

The covariance of the noise is

$$\Big\langle\left[\zeta_y(t) - \langle\zeta_y(t)\rangle\right]\times\left[\zeta_{y'}(t') - \langle\zeta_{y'}(t')\rangle\right]\Big\rangle = \left[r(1-g) + O\left(r^2\right)\right]\delta_{t,t'}\delta_{y,y'}. \tag{45}$$

In the continuum limit, the Kronecker deltas transform as

$$\delta_{t,t'} = \epsilon^k\delta(\tilde{t} - \tilde{t}'), \tag{46}$$

$$\delta_{y,y'} = \epsilon^l\delta(\tilde{y} - \tilde{y}'). \tag{47}$$

Dropping terms of order $O(r^2)$,

$$\langle C(\tilde{t},\tilde{y})C(\tilde{t}',\tilde{y}')\rangle = \epsilon^{k+l}r\delta(\tilde{t} - \tilde{t}')\delta(\tilde{y} - \tilde{y}') + O\left(\epsilon^{k+l+n}\right), \tag{48}$$

where $C(\tilde{t},\tilde{y}) = \zeta_{\tilde{y}}(\tilde{t}) - \left\langle \zeta_{\tilde{y}}(\tilde{t}) \right\rangle$ is defined as in Eq. 24. If we introduce the rescaled noise

$$\eta(\tilde{t},\tilde{y}) = \frac{\epsilon^{-(k+l)/2}}{\sqrt{r}} C(\tilde{t},\tilde{y}), \tag{49}$$

then the covariance

$$\langle \eta(\tilde{t},\tilde{y})\eta(\tilde{t}',\tilde{y}')\rangle = \delta(\tilde{t}-\tilde{t}')\delta(\tilde{y}-\tilde{y}') + O(\epsilon^n) \tag{50}$$

is to highest order independent of $\epsilon$.

### 2.5 Summary

Including only the leading terms, Eq. 24 becomes

$$\epsilon^{k-m}\frac{\partial \tilde{h}}{\partial \tilde{t}} = \epsilon^{2l-m+1}\ln(2)\frac{\partial^2 \tilde{h}}{\partial \tilde{y}^2} - 2\epsilon^{n-m}\tilde{g}r\tilde{h}(\tilde{t},\tilde{y}) + \sqrt{r}\epsilon^{(k+l)/2}\eta(\tilde{t},\tilde{y}). \tag{51}$$

The four different terms scale with the same power of $\epsilon$ if

$$l = \frac{1}{2}(k-1), \tag{52}$$

$$m = \frac{1}{4}(k+1), \tag{53}$$

$$n = k. \tag{54}$$

The inequality of Eq. 43 can be satisfied by $k > 7$. Dividing Eq. 51 by $\epsilon^{k-m} = \epsilon^{2l-m+1} = \epsilon^{n-m} = \epsilon^{(k+l)/2}$ yields Eq. 6 in the main text.

## 3 The derivation of the hull width (Eq. 7 in main text)

We consider Eq. 6 in the main text

$$\frac{\partial h}{\partial t} = D\frac{\partial^2 h}{\partial y^2} - Egh + F\eta(t,y) \tag{55}$$

with periodic boundaries at $y = 0$ and $y = L$. The solution $G(t,y)$ of the deterministic equation

$$\frac{\partial G}{\partial t} = D\frac{\partial^2 G}{\partial y^2} - EgG \tag{56}$$

with initial condition $\lim_{t\to 0} G(t,y) = \delta(y-y_0)$ is

$$G(t,y;y_0) = \frac{1}{L}\sum_{n=-\infty}^{\infty} \exp\left[-(Dk_n^2+Eg)t + ik_n(y-y_0)\right], \tag{57}$$

where $k_n = 2\pi n/L$. The hull position can be expressed in terms of $G$ as

$$h(t,y) = F\int_0^L dy_0 \int_0^t dt' G(t-t',y;y_0)\eta(t',y_0). \tag{58}$$

Combining the last two expression, we can derive

$$\langle h(t,y)h(t,y')\rangle = \frac{F^2}{2L}\sum_{n=-\infty}^{\infty} \frac{1-\exp\left(-2\left(Dk_n^2+Eg\right)t\right)}{Dk_n^2+Eg}\exp\left[ik_n\left(y'-y\right)\right] \tag{59}$$

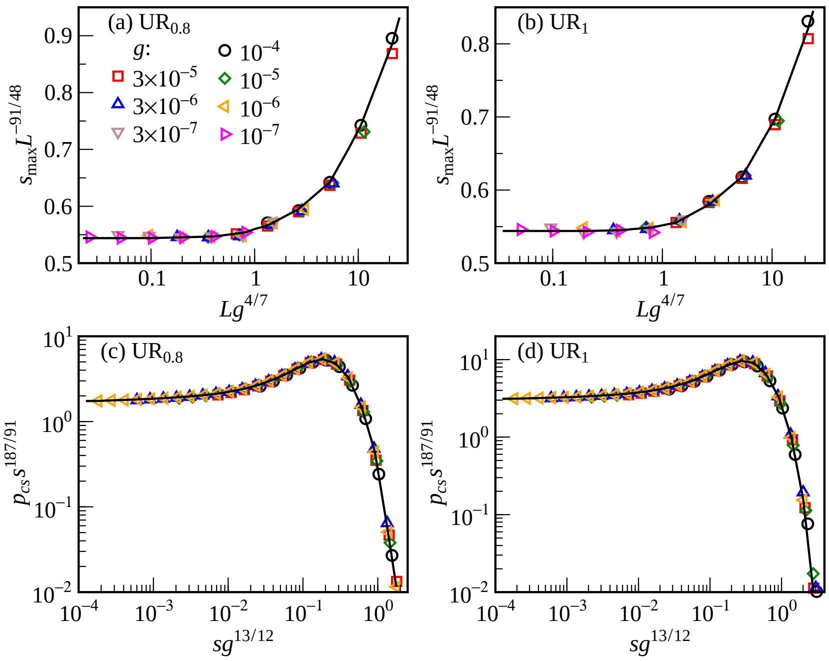


Figure 3: Collapse plots for (a) the size of the largest cluster $s_{\max}$ in $\mathrm{UR}_{0.8}$, (b) in $\mathrm{UR}_1$, (c) the cluster size distribution $p_{cs}$ for $\mathrm{UR}_{0.8}$, (d) $\mathrm{UR}_1$.

The hull width is

$$w^2(L) = \lim_{t\to\infty} \left\langle \frac{1}{L}\int_0^L dy\, h^2(t,y) - \left(\frac{1}{L}\int_0^L dy\, h(t,y)\right)^2 \right\rangle \tag{60}$$

$$= \frac{F^2}{L}\sum_{n=1}^{\infty}\left(Dk_n^2 + Eg\right)^{-1} \tag{61}$$

$$= \frac{F^2}{2}\left(\frac{\coth\left(\frac{L}{2}\sqrt{\frac{Eg}{D}}\right)}{2\sqrt{DEg}} - \frac{1}{EgL}\right). \tag{62}$$

In the limit of large system size,

$$\lim_{L\to\infty} w^2(L) = \frac{F^2}{4\sqrt{DEg}}, \tag{63}$$

which is Eq. 7 in the main text.

# 4 $\mathrm{UR}_1$ and $\mathrm{UR}_{0.8}$ are in the IP universality class

In the main text, we show collapse plots for the maximum cluster size $s_{\max}$ (Fig. 3a and 3b) and the cluster size distribution $p_{cs}$ (Fig. 4a and 4b) for IP and $\mathrm{MV}_1$. In these cases, the data points lie on a single curve when we plot $s_{\max}L^{d_f}$ versus $Lg^{\nu/(\nu+1)}$ and $p_{cs}s^{-\tau}$ versus $sg^{1/[\sigma(\nu+1)]}$. The crucial observation is that the collapse occurs when inserting the IP critical exponents $d_f = 91/48$, $\nu = 4/3$, $\tau = 187/91$ and $\sigma = 36/91$ in these expressions, a telltale sign that $\mathrm{MV}_1$ is indeed in the IP universality class.

In Fig. 3 of this supplement we make the equivalent plots for $\mathrm{UR}_{0.8}$ and $\mathrm{UR}_1$ with the same exponents. The data again fall on a single curve in each case. Moreover, we have already seen in Fig. 2 of the main text that for both of these models $w \propto g^{-\nu/(\nu+1)}$ and $b \propto g^{-1/(\nu+1)}$ as in IP. Thus, all numerical evidence points to both $\mathrm{UR}_{0.8}$ and $\mathrm{UR}_1$ (unlike $\mathrm{MV}_{0.8}$) belonging to the IP universality class.

# References


[1] D. M. Kroll, Z. Phys. B **41**, 345 (1981).

[2] D. D. Vvedensky, Phys. Rev. E **67**, 025102(R) (2003).
